# Visual Perception and the Emergence of Minimal Representation

**DOI:** 10.3389/fpsyg.2021.660807

**Published:** 2021-05-17

**Authors:** Argyris Arnellos, Alvaro Moreno

**Affiliations:** ^1^Complex Systems and Service Design Lab, Department of Product and Systems Design Engineering, University of the Aegean, Syros, Greece; ^2^Department of Logic and Philosophy of Science, IAS-Research Center for Life, Mind and Society, University of the Basque Country, San Sebastián, Spain

**Keywords:** content, cubozoa, minimal representation, neurodynamic structure, perception, vision, constancy mechanism, structural similarity

## Abstract

There is a long-lasting quest of demarcating a minimally representational behavior. Based on neurophysiologically-informed behavioral studies, we argue in detail that one of the simplest cases of organismic behavior based on low-resolution spatial vision–the visually-guided obstacle avoidance in the cubozoan medusa *Tripedalia cystophora*–implies already a minimal form of representation. We further argue that the characteristics and properties of this form of constancy-employing structural representation distinguish it substantially from putative representational states associated with mere sensory indicators, and we reply to some possible objections from the liberal representationalists camp by defending and qualitatively demarcating the minimal nature of our case. Finally, we briefly discuss the implications of our thesis within a naturalistic framework.

## Introduction

Although any representationalist would agree that beliefs, desires and intentions held by humans (or even all primates) are paradigmatic cases of representations, there is a strong debate regarding how far down the line of organismic behavior one is justified to apply the concept of representation. Liberal representationalists (Millikan, 1984, 2004; Price, 2001; Stegmann, 2009; Artiga, 2016, 2021; Ganson, 2020) suggest, more or less, any sensory state that plays the role of a causal intermediary between a stimulus and a behavior should be considered a representation. Such states–usually labeled as mere indicators/detectors–need not be decoupled from their specific stimulus conditions, and the related behaviors can even be highly inflexible^1^. Nevertheless, such states are considered by liberal representationalists to contribute to the explanation of the behavior they trigger in virtue of their putative representational properties^2^.

The non-liberal representationalists (Sterelny, 1995; Ramsey, 2007; Burge, 2010; Rescorla, 2013; Schulte, 2015, 2019; Gładziejewski and Miłkowski, 2017; Shea, 2018) argue that there should be a limit to the range at which the concept of representation could be extended down the line of behavior. They suggest the scope question for the representation should be settled on the basis of whether the representational description of a state has an explanatory value that cannot be attained by a non-representational description of that very state (see Schulte, 2019 for a relevant discussion). Since liberal theories of representation ascribe representational status and properties in states that cannot play a crucial role in explaining a behavior, these theories end up lowering too much the “lower border of representation” (Burge, 2010, p. 549), and they thus face the “breadth-of-application problem” (Burge, 2010, p. 304). Burge’s main argument is that liberal accounts cannot distinguish between sensory registrations and genuine representational descriptions that are associated with a distinctively psychological type of explanation.

Our aim in this paper is to search for the minimal form in which a living being is capable of representing things, properties or states of the environment. According to Burge’s account, perceptions arise as “the most primitive kind of (non-deflated) representation” (2010, p. 316), and such representations are to be distinguished from deflated ones on the basis that they possess contents with associated accuracy conditions. Burge (2010) has strongly and influentially argued that what distinguishes genuine representations from mere sensations, and hence, genuine perception from mere sensitivity, is the employment of constancy mechanisms. However, there are objections. Liberal representationalists (Ganson, 2020) argue that there is no need for the decoupleability introduced by a constancy mechanism for a state to be representational. And some non-liberal representationalists (Gładziejewski and Miłkowski, 2017; Shea, 2018) suggest exploitable structural correspondence is enough for a state to be decoupled from the related sensory stimuli without (even implicitly) considering the necessity for constancy mechanisms in the employment of such correspondence.

In this paper, we aim to contribute to all these controversial questions by complementing Burge in demarcating the minimal case of perceptual representation. We will do so by analyzing (1) what is the minimal form of visual perception, (2) why this perception implies a minimal form of representation, and (3) what is the determinate content of this representation. For the purposes of this paper, we will focus on the visually guided obstacle avoidance in *Tripedalia cystophora* (TC), a small jellyfish belonging to class cubozoa (phylum Cnidaria). We have selected this case for the following two reasons: (i) there is extensive scientific literature on the eyes and vision of this animal, which has been the object of many neurophysiologically-informed behavioral studies (see Bielecki and Garm, 2018 for a review); and (ii) its visual system is considered a minimal but full-fledged form of true vision (perhaps one of the simplest cases of low-resolution spatial vision in nature), a radically new sensory capacity compared to the other classes of Cnidaria.

The paper is structured as follows: in Section “The Earliest Case of True Vision: The Low-Resolution Spatial Vision of the Cubozoan Medusae” we briefly present the optical and visual characteristics of the low-resolution spatial vision of the box jellyfish TC. In Section “Obstacle Avoidance in the Box Jellyfish *Tripedalia cystophora*”, we describe the neuroethology of obstacle avoidance in TC as explored in related empirical studies. In Section “Obstacle Avoidance is Mediated by Structural Representations”, we argue in detail that this case is based on structural representations and is therefore a minimal form of content-based visual perception. In Section “How Minimal are the Perceptual Representations of TC? Replies to Some Possible Objections From Liberal Representationalism” we reply to some possible objections to our thesis from the liberal representationalists camp, aiming mainly to defend and to qualitatively demarcate the minimal nature of our case, while we also briefly discuss the implications of this work within a naturalistic framework. We conclude by summarizing our thesis.

## The Earliest Case of True Vision: The Low-Resolution Spatial Vision of the Cubozoan Medusae

There is a consensus in nowadays science regarding the fact that true vision begins with the formation of a composite set of points in a continuous bi-dimensional part of the eye at which light rays coming from a distal object meet after refraction or reflection, forming an optical image. For a set of light inputs from the surrounding environment forming an optical image at any operational time, the eye detects spatial differences (spatial information) in the form of light levels in the environment and, along with certain neural mechanisms, processes them, discerning lines, shapes and similar features within the field of vision. Thus, the visual system identifies and categorizes environmental features and assesses distances, movements and shapes from among them, which in turn enables body movements to be guided in relation to distal objects. In this sense, vision is *an integration process* of light-based sensory information ending in the formation of a new domain of sensory primitives (i.e., visual percepts) that operate as new irreducible causal factors in the brain, thus enabling new increasingly complex behaviors (Palmer, 1999).

One of the earliest cases of true vision in nature is the low-resolution spatial vision of the cubozoan medusae (see Nilsson et al., 2005; Nilsson, 2009). Cubozoan medusae (also known as box jellyfish) have the most diverse behavioral repertoire of all cnidarians and several of them are light-guided (Garm and Ekström, 2010). Cubozoa are among those jellyfish that respond fastest to changing light levels and object movement through active swimming at rates of up to 2 m per second, combined with rapid turns of up to 180°. And although they possess both mechanoreceptors and chemoreceptors, it is their eyes that enable them to engage in swimming behaviors.

Cubozoa have a squarish (four sides, forming a box) bell that has four complex neural structures called rhopalia located near the edge of each side. Rhopalia harbor the sensorial organs of cubozoa. Whereas other medusae have simple ocelli, cubozoa are unique in the possession of true eyes, which are set in rhopalia. But rhopalia are also the main neural structures of the jellyfish’s nervous system (NS) since they control the swimming muscle contractions.

Each rhopalium contains six eyes, two of which are lens eyes (one located at the lower part and the other located at the upper part of the rhopalium), and the other four are simple pit- and slit-like lensless eyespots^3^. All lens eyes have the major components of a typical camera-type eye, structurally resembling the vertebrate eye (Nilsson et al., 2005, see also Sections “Obstacle Avoidance in *T. cystophora* Involves a Primitive Constancy Mechanism” and “The Characteristics and Properties of the Neurophysiological Process of Obstacle Avoidance”)^4^. The focal length is greater than the distance between the retina and the center of the lens, meaning that light rays are not in focus on the retina, but rather, light from each point in the environment spreads over a large part of the retina thus causing blurred optical images^5^ (Nilsson et al., 2005).

Next, we focus in the most interesting form of visually-guided behavior in cubozoa by briefly describing the neuroethology of obstacle avoidance–driven by the lower lens eyes–in the box jellyfish *T. cystophora* (TC), as explored in related empirical studies.

## Obstacle Avoidance in the Box Jellyfish *T. cystophora*

*Tripedalia cystophoras* are small cubozoa that live in mangrove swamps, where they swim among the prop roots of *Rhizophora* mangle trees passively preying on phototactic copepods which gather in high densities in the light shafts between the roots (Buskey, 2003). The fragility of their one-cell-thick epidermis makes them quite vulnerable in their habitat which is replete with mostly vertical, rough, sharp, or stingy obstacles, some of which are even bacterially contagious for their surfaces. Nevertheless, using its lower lens eyes, TC is able to detect and safely navigate around these obstacles with apparent ease, neither touching nor colliding with them (Garm et al., 2007a).

As any other jellyfish, TC swims thanks to periodic bell contractions which propels it forward. Depending on the patterns of visual stimulation in each one of the rhopalia, discrete motor signals are produced and transmitted to the ring nerve and then through the velarium (a thin muscular sheet) to the bell, constricting it in different directions during swim contractions. When an obstacle appears in front of the animal, its swim pulse rate increases, bell contraction in the direction of the obstacle is delayed, and the opening of the velarium is pocketed out toward a rhopalium in the direction of the obstacle. The result is that the animal turns and swims away from the obstacle with an accuracy of at least 45° in its turning direction (Garm et al., 2007a; Petie et al., 2011, 2013; Garm et al., 2013).

As mentioned in Section “The Earliest Case of True Vision: The Low-Resolution Spatial Vision of the Cubozoan Medusae”, the main task in obstacle avoidance corresponds to the four lower lens eyes, which are continuously active as they scan their distinct visual fields^6^. To analyze this visual process, we will divide the related operations carried out through the animal’s central NS^7^ in three stages. The biggest part of the visual processing happens in the rhopalial NS (see Bielecki et al., 2013 for details). Specifically, in every interaction, the lower lens eye in each rhopalium will process the flow of light inputs and construct an integrated signal in its retina (stage 1). Then, in each rhopalium, the already processed signal from the lower lens eye will be forwarded to the pacemakers^8^ of the rhopalium, where it will be further processed and combined (with other signals coming from the other visual inputs) into one rhopalial output (stage 2). Then, the output signal from each rhopalium will be further combined–through the ring nerve that is responsible for inter-rhopalial communication–with the output signals from the other rhopalia, so as to ensure an altogether efficient response (stage 3)^9^. This will eventually generate the contraction of the bell-shaped body in a discrete (one-to-one) manner that allows the animal to swim (Garm and Bielecki, 2008).

Now, although there are three stages of visual processing, not all these stages and, most importantly, not all related components involved are practically relevant to our discussion about obstacle avoidance. Let us briefly explain why. Starting by the latest stage (3), the fact that rhopalial pacemaker signals trigger a one-to-one swim contraction implies that multiple inputs (i.e., signals from the pacemakers of more than one rhopalium) wouldn’t be effectively functional. The most plausible hypothesis supported by experimental data is that whichever rhopalium is modulated by a visual input at any given time becomes the driving rhopalium that hyperpolarizes the other rhopalia (via the ring nerve), thus resetting the activity of all pacemakers in the other rhopalia. They do so by not only inhibiting them, but also by decreasing them to below baseline, thereby increasing the time required until they can fire again (Stöckl et al., 2011). Thus, the animal’s behavior is practically regulated at each instance of obstacle avoidance by the dominant rhopalium–i.e., the rhopalium whose lower lens eye interacts with an obstacle.

Considering thus only the dominant rhopalium, we pass to stage 2, whose processing happens at the pacemaker. Again, as mentioned, the most likely scenario is that each eye of the rhopalium connects through interneurons to each own pacemaker subsystem, and then the various subsystems are integrated to produce the complex pacemaker signal (Bielecki et al., 2013). However, even if we consider that each light sensory organ produces its own different signal–therefore, some form of necessary integration appears to be taking place within the pacemaker–various experiments have demonstrated that any form of integration in between the pacemaker subsystems will always prioritize the lower lens eye over the other eyes of the rhopalium when the former is currently modulated by visual input with spatial information, producing thus a specific signal characteristic to obstacle avoidance (see Garm and Mori, 2009 for details).

Therefore, and considering only the lower lens eye in the dominant rhopalium, it seems that the most important step in the whole process is stage 1, which happens at the retinal neurons^10^. In typical visual systems, this processing happens in various stages and visual centers. In vertebrates, for example, specific aspects of spatial information (e.g., feature recognition) are handled by parallel pathways of processing that happen in large and complex receptive fields of neurons that are found in higher visual centers than this of retinal photoreceptors (Bartels and Zeki, 1998, see also footnote 20). And these neuronal fields correspond to highly filtered information necessary for the specific visual tasks. The similarity to the vertebrate eye notwithstanding, there are no higher visual centers in TC, since the rest of its central NS doesn’t have the resources to process spatial information coming from the lens eyes (Skogh et al., 2006). However, spatial information sensitive to the task is processed directly by the retina: large receptive fields are found at the level of retinal photoreceptors, each one of them allowing complex filtering of different aspects of spatial information in parallel, much earlier than in more advanced visual systems (Nilsson et al., 2005). Let us see how.

There are at least two large and complex receptive fields at the level of retinal photoreceptors in TC that correspond to the so-called matched filters that enable TC’s NS to deal only with environmental information essential to the task. The first one enables a low-pass spatiotemporal filtering. This gives a spatial resolution of 10°–20° (depending on the retinal area), and a low temporal resolution for the lower lens eye (O’Connor et al., 2010). The second matched filter enables pattern-dependent sensitivity and directionality of the response to the task. As we will see in more detail next, a series of behavioral experiments have shown that the animal is more able to detect vertical than horizontal obstacles. This is probably due to the fact that the retina of TC is genetically better at detecting more vertical than horizontal contrast line orientations due to directional contrast enhancement^11^. The combination of low-pass spatiotemporal filtering and pattern-dependent directionality removes informational aspects that are irrelevant to the context of obstacle avoidance and allow the essential ones to be further processed by the NS. The combination of these two matched filters enables the animal to detect large and stationary/slowly moving and mainly vertical structures strongly resembling the prop roots of the mangle trees among which it swims in search of light shafts full of copepods^12^.

In the lack of further processing by higher visual centers in TC’s NS, this combination of the two matched filters cannot be implemented without some form of integration^13^ at the retina, which will produce the unique visual signal to be sent to the related pacemaker subsystem, so that stage 2 of processing can begin. Matched filters work by extracting the sensory stimuli crucial for the animal’s survival, severely limiting the non-important stimuli according to the task (Warrant, 2016). In the next section we argue in detail that obstacle avoidance in the box jellyfish during stage 1 of visual processing is a minimal form of content-based perception.

## Obstacle Avoidance Is Mediated by Structural Representations

A series of behavioral experiments by Garm et al. (2007a, 2013) has shown that obstacle avoidance is indirectly correlated with the size of the obstacle on the retina, based on actual distal detection of the obstacle as an object in an image using spatial information that is both contrast- and pattern-dependent. In their experiments Garm et al. (2013) found, as a general result, that TC will initiate obstacle avoidances more frequently and further away from the obstacles the higher the contrast of the obstacle and the closer to vertical its orientation (*ibid.* Figures 1–3, pp. 4,521–4,523)^14^. This task involves processing a lot of spatial information (with several important aspects relevant to the task, such as size, orientation of the object, and distance to the object) for a limited number of neurons in the rhopalium.

The experiments have also shown that if the eye is triggered by a visual scene with no spatial information (i.e., lack of a simultaneous combination of light rays coming from different directions associated with spatial differences) but with a uniform decrease of the same magnitude in the light intensity across the entire visual field (such as that produced in the presence of an obstacle), the animal fails to elicit any obstacle avoidance behavior. This demonstrates that the animal detects the obstacle as an object using spatial information (Garm et al., 2013, Figure 4, p. 4,524)^15^.

Based on these experiments, in the following subsections we will discuss in detail three main aspects of this visual process: (i). that the visual state that guides obstacle avoidance employs a constancy mechanism, (ii). the integrated nature of the retinal processing that decouples the visual state from its environmental antecedents, and (iii). the similarity of the visual state to its target object. Although we treat these three aspects separately for demonstrating purposes, it should be noted that they are different aspects of the same neural structure, namely of the formation of the visual state that guides obstacle avoidance. As we explain in Section “The Representational Role of the Neurodynamic Structure of Obstacle Avoidance”, these three aspects of visual processing are all together necessary and sufficient for a visual state to be representational.

### Obstacle Avoidance in *T. cystophora* Involves a Primitive Constancy Mechanism

In the experiments by Garm and colleagues, the mean size of the obstacle on the retina capable of eliciting a response (i.e., the minimum angular distance between two objects required for the animal to see them as two rather than one merged object) is around 25°, and this is almost the same for any object with highest contrast (Garm et al., 2013). So, if the object is around 2 cm wide and within 5–6 cm^16^, TC will detect and try to avoid it (*ibid*, Table 1, p. 4,526). Interestingly, the experiments demonstrate that TC won’t elicit an avoidance response when it is confronted with 2 cm-or-wider objects that are far away, but it will avoid objects down to 1 cm wide if they are very close (Garm et al., 2007a). This shows that the eye is not just a simple trigger sensor, which would yield a non-differential obstacle avoidance to all 2 cm-or-wider objects in the environment, incapable of avoiding obstacles very close to the animal. On the contrary, the lens eye of TC is able to accommodate such foraging-related capacities with the employment of a very primitive (or pre-) constancy mechanism that provides an ecologically related stability to the animal’s decision to initiate obstacle avoidance. Let us elaborate a bit more on this.

The animal depends on the flow of visual sensorial registrations to successfully control its swimming with respect to the target object–i.e., to regulate direction and speed while remaining far away from the object. And distal actions would likely require correlating with environmental properties relevant to the task–in our case, a certain width and orientation of the object so that it resembles a prop root to be avoided. Yet, as Burge has pointed out (see Burge, 2010, pp. 397–398), these crucial (to the task) aspects that are conveyed by sensory registration underdetermine the object properties, since they are also influenced by the circumstances of the interaction (perspectival aspects) –in our case, the TC’s positioning in the environment. This part of the sensory registration influenced by the perspectival aspects is not likely to correlate with environmental conditions relevant to the task, thus causing variation and ambiguity to the proximal stimulations (to be processed by the retina) with respect to the properties of the object. Therefore, a putative constancy mechanism in TC should at least provide a form of stability in the production of percepts of obstacles to be avoided in the face of ambiguous variations in the proximal stimulations of the target object due to changes in TC’s positioning with respect to that object. How could the lens eye of TC do that?

One aspect of the experiments by Garm et al. (2013) that is particularly worth noting is that there seems to be a contrast-dependent mechanism for detecting the distance of an object in the visual scene^17^. Due to its blurred vision and relatively scarce neural resources, TC does not have the means to precisely determine distance. But the animal uses instead its ability to measure the light contrast of a given object, which decreases with distance in the turbulent waters in which it lives due to high light absorption and scattering. This implies that TC uses contrast to indirectly detect the distance of an object (see *ibid*, p. 4,526). This is crucial for a putative constancy mechanism. More specifically, and applying the two-track account of gradual constancy suggested by Schulte (2020), the primitive constancy mechanism in TC can be viewed as being crucially sensitive to a particular proximal variable, namely the *size of the object’s registrations on the retina*, which is causally dependent on both the obstacle size (the target variable) and the distance from which the obstacle is seen (the confounding variable)^18^. What “disentangles” the information about an object’s size contained in retinal registrations from the information about its distance is that the visual mechanism is sensitive–although, in our case, mostly for vertical objects–to another (auxiliary, and in this case proximal) variable, namely contrast, that acts as an indirect cue^19^ used by the animal to calculate the distance to the target object. And this is why, most of the time, TC manages to respond differentially to the ecologically relevant size (and distance) of objects (i.e., to potential obstacles) and not merely to the direct information of their registrations on the retina.

### The Characteristics and Properties of the Neurophysiological Process of Obstacle Avoidance

As we have seen, visually guided obstacle avoidance requires processing a lot of spatial information. In the vertebrate eyes, the environmental stimulations registered on the retina are further filtered and processed in parallel retinal pathways and then integrated into a unique visual signal^20^. We have no specific neurophysiological knowledge of how optical information from the lens eyes of box jellyfish is processed by the retina (Bielecki and Garm, 2018), and we don’t expect the same degree of functional complexity (and of the underlying circuitry) in the retina of TC. However, apart from the resemblances regarding the optical characteristics, the essential architecture and functioning of the retina, as well as the configuration of the eye in the NS of TC–practically, the eye is directly embedded in the central NS–is similar to the situation in vertebrates (Garm et al., 2006). As such, in the context of parallel retinal processing, the term “integration” could be broadly understood as a phenomenon that occurs when a set of different and initially independent processes–such as those of the two matched filters–functionally cooperate and share their local functions, leading to the establishment of a wider functional organization, in which some functional constraints of the constituent processes are interlocked and control each other, producing thus a unique visual signal. The emergence of a new functionally integrated structure in the retina, therefore, requires a functional redefinition of the original processes of extraction of the relevant sensory stimuli and of the eventual matched filtering.

In the context of retinal processing, the role played by single neurons cannot explain a functional motor action like distal swimming. Neurons can change the state of the NS, but they are still cut off from direct action in the outside world, being insulated from it by other processing groups of neural cells that lie between them and the muscles. So, the function changes across levels (Cao, 2012). Thus, in the case of distal obstacle avoidance, only a higher and integrated level of the retinal information can generate a meaningful set of functions. Accordingly, in TC, the continuous sensory information registered in the retinal receptors is received, filtered and processed in parallel, and eventually integrated to form a temporally discrete *neurodynamic structure* (NDS)^21^, with a clear functional relevance in visually guided swimming. It is this NDS that drives the distal avoidance of objects.

Therefore, by the integration of a continual flow of the registered retinal data, a NDS in cubozoan retinas acts as *decoupled and higher-level control*, guiding obstacle avoidance in a context of uninterrupted sensorimotor interactions of the box jellyfish with the environment. The integration processes at the retina introduce a decoupling of the retinal state from the environmental stimulus, since the functioning of the NDS cannot be reduced to the functioning of single retinal neurons nor to any set of them. This decoupling of the NDS becomes apparent also through the delay of its formation. While the animal incessantly registers stimulations in its retinal cells as it swims and visually interacts with its natural habitat, these stimulations cannot be integrated into a visual signal by its eye in less than just over 100 ms (see O’Connor et al., 2010). So, there is a neurophysiologically considerable delay in the formation of the NDS in relation to the continuous registration of environmental stimulations due to the integration processing in the retina.

### Successful Obstacle Avoidance Depends on Structural Similarity

The behavioral assays by Garm et al. (2013) showed the following results regarding TC’s interaction with obstacles of different contrast and orientation:

1. Increasing contrast results in the animal staying farther away from the obstacle and initiating more avoidances for all three stripe orientations (*ibid*. Figure 6, p. 4,527).
2. Contrast-dependency varies in accordance with the orientation of the stripes, with the animal having the strongest response to obstacles with vertical stripes, an intermediate response to those with oblique stripes, and the weakest response to those with horizontal stripes, for all different contrasts (*ibid.* Table 1, p. 4,526).
3. There is a gradual change in the strength of the response (measured through the average distance from the obstacle, avoidance rate, and obstacle size on the retina), at least for those obstacles with vertical stripes (*ibid*, p. 4,526).

When the box jellyfish is close to an object in its environment, its capacity to distinguish that object as a full and separate entity is directly proportional to its contrast, size, verticality, and immobility, and secondarily (and indirectly as discussed) to its size. Otherwise, so long as the animal, for whatever reason, cannot visually detect an object with respect to its size, shape and gray-toned color (i.e., it cannot distinguish its spatial information from the rest of the visual scene), obstacle avoidance is poorer (in terms of avoidance rate and distance from the object). Moreover, there is a gradual change in the strength of the response (avoidances happening more frequently and farther away from the obstacle), with vertical shapes initiating stronger avoidances, followed by oblique and then horizontal ones. In other words, the darker, larger, slower, and more vertical the obstacle is, the faster and more frequently the animal detects it as a full and separate object, and the faster and more frequently it will avoid it. When confronted with a dark, relatively large and vertical object, should the animal, for whatever reason (water murkiness, turbidity, etc.), construct a different (*and less similar*) NDS (e.g., a retinal integration producing a combination of patterns of neuronal activation corresponding to an oblique, light gray object), this would result in less successful obstacle avoidance, since the response would be initiated not only less frequently, but also more slowly, i.e., at a smaller distance from (closer to) the object.

We could infer from these behavioral data, that this is a case of *structural similarity* between the visual percept and its target in the environment. It is the global structure of the retinal organization (NDS), whose integrated sub-patterns of activation are associated (at least) with size, contrast, and shape, that corresponds to objects of a certain size (and shape) at a certain distance in the environment. So, when the correspondence between components in the visual percept (sub-patterns in the integrated retinal organization) and size, distance, and shape of the objects in the environment is strengthened, the likelihood of TC achieving obstacle avoidance increases, and vice versa^22^.

This systematic correspondence between the relevant features of the environment and the NDS formed by the lower lens eye, as well as the relationship between this eye and the other visual sensors (stage 2 and 3 of processing), is deeply embedded in the animal’s context of action (i.e., on what the animal is doing and the type of environment in which it is doing it). The lower lens eye will be involved in obstacle avoidance whenever a full object is detected in the retina, but it will be involved differently (or not at all) when there is simply a change in ambient illumination, since in that case the animal is likely to be in a light shaft containing swarms of copepods to be caught and eaten. Therefore, the NDS of the lower lens eye during obstacle avoidance exhibits a considerable degree of exploitable structural similarity to its target in the environment (see Gładziejewski and Miłkowski, 2017; Shea, 2018). Moreover, the homomorphism between the NDS and the world preserves not only the static structure of the object, but it also preserves a resemblance between the different NDSs and the downstream processing in which the obtained similarity plays a causal role. And this is evident by the fact that it’s exploitation by the animal provides differential responds to the world that hold the said resemblance (i.e., a set of like objects at nearby locations in the environment will generate the same or closely related responses).

We conclude that we can systematically correlate similarities between changes in the environmental structure and changes in the animal’s obstacle avoidance behavior on the basis of the exploitable structural similarity between the NDS and the related environmental structure. Also, the similarity does neither obtain at the level of each cellular state nor at any range of them as they are reflected on the retina and construct an optical image (due to integration processing at the retina). Moreover, the homomorphism provides also a similarity between possible stimuli and possible responses (due to exploitation). Accordingly, the NDS in TC constructs a second-order structural similarity to its target object (see also Isaac, 2013; Piccinini, 2018). We suggest this is the most sensible explanation for the NDS at the retina of TC based on the available neurologically-informed behavioral data regarding the retina’s workings and its relation to obstacle avoidance.

### The Representational Role of the Neurodynamic Structure of Obstacle Avoidance

Considering all these aspects together, there are good reasons to defend that the cubozoan visual system implies a minimal form of representation. Next, we will briefly present these reasons.

In order to legitimately consider something–a state or structure–a representation, the general consensus is that it must fulfill three conditions. Let us primarily consider the first two: (i) that a representation should cause successful actions, and (ii) be decoupled from the structure of the related incoming physical inputs (i.e., light rays captured by the animal’s eyes). In other words, to be representational, the state or structure cannot be a mere indicator^23^. In relation to the first condition, we have shown that TC’s distal action is based on the NDS (the neural support of the visual percept), hence the latter plays an essential role in successfully avoiding obstacles. The second requirement is also fulfilled. Although Orlandi (2014) claims that minimal vision is not representational because there is no capacity for internally recording absent things, she nevertheless considers the capacity to perceive an entity which is only partially seen to be an instance of minimal absence (see pp. 125–127)^24^. Certainly, the cubozoan NS does not yet have the capacity for storage (memory) and associative learning required for off-line use of the distal information provided by visual percepts. However, what makes the swim guiding NDSs representations rather than mere indicators is that their construction and modification/updating (a) are endogenously controlled; (b) depend on an internal integrative organization of the NDS itself, and (c) employ a primitive constancy mechanism so that they are not determined (although they may be affected) by the causal coupling with the target. Taken together, all this may be considered sufficient indication that the NDS does its job “in a decoupled way” from its target. Thus, the NDS that guides obstacle avoidance in TC is a performance-guiding structure that is decoupled from environmental light inputs (generating continuous sets of microscopic retinal stimulations) because it is the result of a complex process of integration that employs (primitive) constancies.

These factors are precisely what lead Burge (2010, 2014) to argue that visual perception is a minimal form of representation. He further argues that since the construction of a visual percept is underdetermined by the raw visual data, solving this problem–by prioritizing certain possible environmental (distal) causes over others–leaves the formation of perceptual states vulnerable to error. Yet, we think Burge hasn’t realized this is only a necessary, not a sufficient condition. And this is what drives us to the third and fundamental requirement for a structure to be considered as a representation, emphasized by many other authors. O’Brien and Opie (2004), Isaac (2013), Gładziejewski (2016), and Gładziejewski and Miłkowski (2017), for instance, argue that a state or structure can be legitimately considered a representation only if there is an (exploitable) structural similarity/correspondence between the candidate structure and the environmental features that are represented (i.e., the target of the representation). We agree with these authors on this point, since only in such a relational context (such as that of structural similarity) successful distal action requires the capacity to construct NDSs which are (structurally) similar (to some extent) to some conditions that depend on a specific set of environmental elements relevant for the target action. It is only in this context that it would be logical to assume that any deviation from the required similarity will diminish the success of the action.

And this is where accuracy and error come non-derivatively into play, since it is only within a context of a “correspondence”–between the NDS and its target in the environment–that ignoring and detaching from the continuous proximal registration that underdetermines the NDS can be considered to have accuracy conditions with respect to the environmental causal antecedents of this very structure. In turn, it is only in such a context that it could plausibly be suggested that inaccuracy (or misrepresentation) occurs–because of an ecologically-significant mismatch between the structure of the target domain and the actual action-guiding structure of the representation (see also Lee, 2018, p. 616).

Structural representations cannot misrepresent on their own; they either function or not. But as we discussed, the perspectival nature of TC’s visual interaction implies that structural representations induced by the same causal process may contain very different quantities of information about the target. Of course, as Burge generally states, constancy is exactly the mechanism to avoid such errors resulting from the underdetermination of the object by the sensory stimulation. However, the employment of any constancy mechanism would be totally “blind” to the task had the retinal processing pathways been hardwired (through evolution) to construct a NDS that doesn’t hold any structural resemblance to its target in the environment, or (most importantly) had it been hardwired in a way that (most of the ecologically-relevant times of the interaction) the eye wasn’t able to construct a “similar” structure. So, it’s not that constancy mechanisms are not necessary for the possibility of error, but, more correctly, that constancy mechanisms would be relevant to the task (and hence, prone to error) only if they are employed by a sensory state that constructs structures through which it can successfully interact with the environment^25^. Accordingly, a neural structure bears content, in so far as it is a NDS whose role in the organism’s capacity for adaptive interaction depends on its accuracy in the context of similarity/correspondence. And this is why the NDS has the content it does; it is precisely because of its similarity to environmental factors that it has conditions of accuracy^26^.

Summing up, we have explained (i) how the cubozoan NDSs of the lower lens eyes possess content; and (ii) how–and why–these NDSs play a causal role by virtue of their content. This latter aspect is a fundamental point because, as Ramsey (2007) argues, for a structure to be representational it is required that its content would serve or function as a representation in a larger system (see also Hutto, 2011; Segundo-Ortin, 2019). This structure must be shown *to play a causal role in the organism’s functioning by virtue of its content*. This is the aspect of “why” that Ramsey emphasizes. In other words, something is representational when its similarity to a state of the world not only “explains” a successful action, but also causes it. In our case, answering to Ramsey’s question, namely, why the NDS of our case is representational, we reply that it is so because it enables the animal to act from a distance by virtue of the accuracy of its NDS. Only if the structural similarity between the NDS and its target–obstacles in the environment–is causally relevant to the success of the mechanism (i.e., obstacle avoidance) that makes use of the representation can we ensure that “Ramsey’s job” is fulfilled.

## How Minimal Are the Perceptual Representations of TC? Replies to Some Possible Objections From Liberal Representationalism

In what follows we comment on a set of possible objections to our thesis coming from the field of liberal representationalism^27^. Liberal representationalists defend a very extensive use of representations, where some internal states of very simple organisms (like unicellular organisms or plants) are considered as genuine representations (Artiga, 2016, 2021). We certainly disagree with this position, since, as Ramsey (2007), Burge (2010), Orlandi (2014), and Gładziejewski and Miłkowski (2017) among others suggest, liberal and deflationary accounts can easily fall prey to panrepresentationalism, or/and trivialize and dissolve the notion of representation to the point that it abolishes any explanatory power. Yet, here we will not discuss this issue in general but only in what affects our thesis, since according to this position, our case study would certainly be an example of representationally guided behavior, but not a minimal case.

In a recent paper Ganson (2020; see also Artiga, 2021 for a similar view) argues that representations do not require decoupling from specific stimulus conditions. Ganson’s point is that the information-carrying role of sensory states is required to explain successful task performance, i.e., successful coordination of outputs with environmental conditions conducive to the success of those outputs. For example, the coordination of ovulation with the presence of a male in the phenomenon of induced ovulation through detection of pheromones. In this case, Ganson argues, the stimulus condition (e.g., pheromones) is different from the environmental condition with which the animal’s behavior should be coordinated (e.g., a potential male). For the coordination with the environment to be achieved, the potential information about significant features of the environment (potential male)–carried by the stimulus condition (pheromones) and exploited by the receptors–should be added to the causal chain of events from stimulus to behavior. Hence, sensory states have an information-conveying role in addition to their causal role, and we can separate the former from the latter by intervening on the former (e.g., by manipulating the environmental conditions). These interventions, according to Ganson, reveal the explanatory role of information in successful task performance. Accordingly, the success of the organism’s inflexible response to the stimulus depends on the informational content (instead of the stimulus) of the sensory state. In general, liberal representationalism argues against the requirement of decoupleability in order to distinguish the merely causal from the representational role of sensory states in highly inflexible behavior.

But if all correlational information necessary for the behavior’s success could only be altered and manipulated externally, then the outcome of the behavior wouldn’t be explained by the information-carrying role of the sensory state but by the environment itself. Accordingly, the coordination of highly inflexible behaviors guided by sensory states triggered by stimulus conditions that are not present in the environment seems like (or are even weaker than) a mere reaction–if not a blind guess–on the part of the organism. Hence, it is highly undetermined how those sensory states come to do any explanatory work, at all. The problem is not so much that those sensory states are dispensable to the explanation of the related behaviors (the sensory state does causal work–what Dretske (1988) calls a “structuring cause”), but that there is no content, at all–at least not on the behalf of the organism.

Yet, as we will explain, the requirement for constancy-dependent decoupleability (and consequently, for the endogenous control of the construction of the representational state) is also crucial in explaining the qualitative difference between structural representations and mere indicators/detectors. Take for instance the escape behavior of the hydrozoan *Aglantha digitale*. Lacking vision, *A. digitale* relies on many different proximal sensors. When a predator approaches, it produces a certain type of vibration in the water that activates the animal’s hair-like sensory cells. These cells thereupon send a spike directly to the ring giant axon, which is propagated in both directions all along the ring, activating eight motors, thus driving the animal upward very quickly. The general feature of the different behaviors displayed by *A. digitale* (as well as by other swimming medusae lacking true vision) is that, in each case, a sensory detector (or a combination of different sensory detectors) directly converts proximal stimulations into signals for the animal’s NS (Mackie, 2004). Liberal representationalists would argue that since the proximal registration (the vibration of the water) produced a certain sensory state (the stimulated hair-like sensory cells) causing the giant ring axon to fire and thus contracting the muscles and enabling the animal to swim in direction different to the vibrations, the sensory state is representational. However, in the absence of any constancy mechanism, this would be explanatorily unnecessary. Since, in the case of purely biological behaviors, the internal states triggered by the stimuli do not play any causally efficacious work apart from its structuring cause, ascribing them a representational status would be explanatory dispensable with respect to the behavior. Let us elaborate on this.

The triggered state indicates movement in the water (the distal target object). Now, if the sensory system of *A. digitale* were isomorphic to the vibrations of the water (i.e., for every change in the water there exists a behaviorally significant change in the animal’s sensors, and vice versa), then the sensory state would operate as a switch specific for each instance of vibration (i.e., for each different value of the target object). In this case, *A. digitale* could react in only one specific (and possibly different) way for each one of the different instances of water vibration, independently of the animal’s perspective. Presumably, the sensory system has a homeomorphic relation to changes in the water (i.e., for a behaviorally significant change in the animal’s sensors there exists a change in the water vibrations, but not vice versa), and the sensory system is capable of discriminating ranges of vibration strength (e.g., from a weak vibration produced by a small rock hitting the water surface to a strong vibration produced by the movement of a big fish nearby). In this second case, *A. digitale* can again react in only one (and again, possibly different) way for each value of vibration within the various different ranges of water vibration (e.g., keep swimming leisurely in case of a weak vibration produced by a small rock, and escape in case of a strong vibration produced by a big fish), but again, independently of the animal’s perspective. So, in both cases, *A. digitale* would be able to produce always the same (though possibly different from each other) behaviors for each one of the different (ranges of) instances of the distal target object. And this is why, in both cases the *A. digitale’s* sensory system is capable only of inflexible behaviors. Accordingly, none of the aforementioned biological facts regarding the escape circuitry has any representational role to play in the explanation of *A. digitale’s* behavior, exactly because, due to the inflexibility of the behavior, there is no space for any causally efficacious meaning of the sensory state after the description of the complete causal chain of events from the stimulus to the behavior^28^.

Things are substantially different in TC. Firstly, the sensory system in TC enables the animal to deal in the same way with different instances of the distal target object–the animal will avoid objects of different shape at the same distance or/and the same object at the same distance when it sees it from different angles. But most importantly, TC will demonstrate different behaviors for the same instance of the distal target object (i.e., a prop root with a certain size and shape), depending on the animal’s perspective (its distance to the prop root). Therefore, in stark difference to the sensory system of *A. digitale*, the capacity of the lower lens eye in TC to build a constancy-dependent percept (supported by the NDS) is what explains its capacity for displaying actively flexible interaction with the same instance of a target object in a range of different contexts/perspectives. This is why, contrary to the case of *A. digitale’s* escape (or any other kind of similar) behavior, the NDS of the lower lens eye does added work in the explanation of TC’s obstacle avoidance behavior. And it does so because the NDS is what explains the animal’s choice among different causal chains (i.e., available options) for its interaction with the same instance of a distal target object, depending on the context of the interaction–i.e., on the animal’s perspective. In the case of purely biological behaviors, the internal states triggered by the stimuli do not play any causally efficacious work apart from its structuring cause, hence ascribing them a representational status would be explanatory dispensable with respect to the behavior. Additionally, this is why not only constancy mechanisms need a context of structural similarity in order to produce veridical content, but also, it is the reason why structural representations require constancy-dependent decoupleability, so that their difference to indicators/detectors cannot be considered as one of a degree (as suggested by Nirshberg and Shapiro, 2020) but of a kind.

Therefore, as we have argued so far, there is a significant difference between a constancy-bearing structurally similar state and a mere indicator. And this can also be illustrated in a complementary way applying Sterelny’s distinction between robust-process and actual-sequence explanation (Sterelny, 1995; see also Schulte, 2015). In general, any behavior can be explained by a careful description of the sequence of the actual events. Obviously, both the cases of *A. digitale’s* escape and of TC’s obstacle avoidance can be explained by the description of the precise sequence of the actual neurobiological events that led to the motor actions expressed in the behaviors of the two animals. But the case of TC can only be completely explained on the basis of a degree of similarity between the NDS and the distal features of the environment, as a result of being guided by a sensory (visual) state elaborated by constancies. And this explanation would be valid for a range of environmental conditions (e.g., for objects of different sizes, seen from different distances or/and angles, with different contrasts). In the case of *A. digitale*, the actual-sequence type of explanation is also the most robust-process type of explanation available, whereas in the case of TC, a robust-process explanation is qualitatively different than the actual-sequence one, since it is an explanation freed from the specificities of the proximal retinal stimulations, and therefore, it is an explanation that includes the various options of behavior available to the animal when it interacts with the same object. And this is possible because obstacle avoidance is a (genuinely) intentional behavior targeted to distal features that are represented by the animal.

So, constancy-dependent decoupleability within a context of structural similarity of a neuronal state with its environmental target provides a qualitative distinction between structural representations and mere indicators because it offers a connection between genuinely intentional explanations and their neurodynamic underpinnings. In other words, the kind of decoupleability described in TC relates an intentional explanation of the animal’s behavior with the causal role played by the NDS in guiding this behavior by virtue of its content.

## Conclusion

In the quest for demarcating the minimally representational behavior, we provided “flesh and bones” to Tyler Burge’s influential claim that the limits of genuine representational behavior are the limits of perception satisfied by the most primitive (non-deflated) representational states. Burge has strongly argued that what distinguishes genuine (non-deflated) representational states from mere indicators is the employment of constancy mechanisms. However, other non-liberal representationalists have argued that a considerable degree of exploitable structural correspondence of a state to its target is sufficient for a state to bear content. Complementing Burge, we have argued that it is only within a general context of similarity/correspondence of a constancy-bearing neural structure that veridical content arises.

We focused on perhaps one of the simplest cases of behavior based on low-resolution spatial vision in nature–the visually-guided obstacle avoidance in the cubozoan medusa *T. cystophora*–and we argued in detail that this is a case of a minimal form of content-based visual perception. More specifically, and based on the neurophysiologically-informed behavioral data available in the literature, we explained that the lower lens eye in TC employs (primitive) constancy mechanisms to construct a decoupled (from the retinal stimulations) neurodynamic structure, based on which it succeeds obstacle avoidance in virtue of this structure’s exploitable structural similarity with its target objects in the environment. Considering that the lens eyes of TC are the most basic case of true camera-type eyes in nature, obstacle avoidance in TC is guided by a minimal form of perceptual representation.

Through this case study, we have shown the operational difference between genuine representational behavior and reactions based on mere sensations. Based on an analysis of the empirical data of the obstacle avoidance swimming of TC, we have also argued how this behavior is based on the construction of an integrated neurodynamic structure using a primitive form of visual constancies. This in turn shows a clear form of decoupleability from the raw sensorial registration captured by the visual organs of the animal. Last but not least, we have argued in what sense the visual percepts of TC show a form of structural similarity with its environmental target.

All this provides a qualitative distinction between structural representations and mere indicators, offering also a connection between genuinely intentional explanations and their neurodynamic underpinnings. And this, we suggested, should be indicative of the qualitative distinction between intentional/cognitive and purely biological behaviors–an aspect that must be much further pursued by a theory of minimal representations.

## Data Availability Statement

The original contributions presented in the study are included in the article/supplementary material, further inquiries can be directed to the corresponding author.

## Author Contributions

Both authors contributed to the article and approved the submitted version.

## Conflict of Interest

The authors declare that the research was conducted in the absence of any commercial or financial relationships that could be construed as a potential conflict of interest.

## Footnotes

1 Characteristic minimal such cases are any kind of taxis, such as chemotaxis, phototaxis, and magnetotaxis, etc., even in unicellular organisms, as well as in plants and earthworms.

2 However, not all liberal representationalists adhere to such a deflationary account. Dretske (1986, 1988), for instance, distinguishes between recruitments done in biological organisms via natural selection and others done via some kind of learning. In these latter categories, behavior can be altered based on the manipulation of the information carried by the conditioned stimulus. In such cases, the organism’s behavior depends on the information carried by the related sensory state, hence, the state is more than a causal mediator in the relation between the organism and the environment; for Dretske, it carries information in a way that it should be considered a representation.

3 These eyes are adapted to different elements of the visual environment and perform different visual tasks (Land and Nilsson, 2006). Because of the configuration and the orientation of the rhopalium, only the lower lens eyes are involved in obstacle avoidance (Garm et al., 2007a). Due to its position, the upper lens eye is used for long-distance navigation rather than obstacle avoidance (Garm et al., 2011). The function of the eyespots is very little understood, but it seems that they are not involved in the sensorial information relevant to obstacle avoidance (Garm and Bielecki, 2008).

4 There is a cornea (a covering layer of transparent cells), a crystalline lens with a refractive index gradient, a layer of dark pigment covering the outside of the retina and forming an iris around the lens, and a retina filled with microvilli that protrude from the outer ciliary segments of its photoreceptor cells (for details see Nilsson et al., 2005; Land and Nilsson, 2012, pp. 76–77).

5 See Figure 4.4c in Land and Nilsson (2012) for a computer modeling of the blurred portrait of one of the authors as it would be constructed by the lower lens eye of the box jellyfish *Tripedalia cystophora*. According to Seymour and O’Hara (2020), the under focused vision of *T. cystophora* is not an intrinsic limitation of cubozoan vision, but a relationship between visual acuity and feeding ecology. For example, the cubozoan *Chironex fleckeri* has a more acute vision because it feeds on fast agile prey fish (whose locating requires sharp image focus), which is not the case of *T. cystophora* that feeds on dense copepod swarms and therefore does not need to visualize fine details while hunting (see also footnote 12).

6 In obstacle avoidance–as in all types of TC’s visual interaction with the environment–all other (upper) lens eyes as well as the eyespots are also simultaneously and continuously active receiving light rays from their own visual fields and sending signals.

7 The central NS of TC comprises the photoreceptors of the retina, the four rhopalial nervous systems, and the ring nerve (Garm et al., 2006).

8 A pacemaker is a set of subsystems of neurons that produce the discrete motor signals that are sent to the muscles and control the animal’s movement via bell contractions. Cubozoan pacemakers are located close to the insertion of the rhopalial stalk (Garm et al., 2006).

9 Each rhopalial NS is directly connected to the ring nerve through a bidirectional epidermal stalk nerve through which each rhopalium is also attached to the bell of the animal (Garm et al., 2007b).

10 Indeed, in TC, the synaptic morphology of the photoreceptor cells in the retina, as well as the whole rhopalium, suggests that the first stages of visual processing occur in the retina (Gray et al., 2009).

11 The morphology of the synapses between neighboring photoreceptors in the lower lens eye suggests enhancement of vertical stripes (Gray et al., 2009).

12 The fact that the lower lens eye has a specific and heavy spatiotemporal filtering at the periphery is vital to efficient obstacle avoidance, since it prevents responses from interfering with the animal’s (much faster though blind) foraging behavior–the animal is blind to the small and fast-moving copepods, which it hunts only by the touch as it swims in the light shafts with its tentacles spread.

13 In the next section we develop this point.

14 The authors presented the animal with vertical obstacles with three different stripe orientations (vertical, 45° oblique, and horizontal) set against five different gray tones, resulting in different contrasts ranging from 0.93 (the darkest) to 0.17 (the lightest).

15 This has also been electrophysiologically tested by projecting of moving black/dark bars (resembling vertical obstacles) traveling across the animal’s visual field. This resulted in a sharp increase in the swim pacemaker signaling frequency, while a uniform decrease of the same magnitude in light intensity across the entire visual field triggered no response at all (see Bielecki and Garm, 2018, Figure 27.10, p. 618).

16 A visual angle around 25°–30° corresponds to obstacles of a diameter of around 2 cm in a distance of 6–5 cm (Garm et al., 2007a).

17 As we discuss in the next section, this doesn’t mean that obstacle avoidance in TC is triggered by a contrast threshold (Garm et al., 2013).

18 Drawing on Schulte, a constancy mechanism can be distinguished from a mere sensory indicator by the fact that it is based on information of two variables. In our case, the variable (K) of the object’s size depicted at the retina is “informationally ambiguous” not only in the general sense that a particular value of K (i.e., a particular size of the object’s depiction at the retina) is compatible with many different values of T (i.e., many different object sizes when viewed from different distances), but also because, under biologically normal conditions, not all activations of 2 cm or more at the retina should evoke an avoidance. Hence, values of K do not carry any biologically significant amount of information about the value of the target variable T unconditionally (see 2020, pp. 12–13 for a detailed discussion).

19 We say this is a primitive case of a constancy mechanism because (i). the stability in the veridical percept is enabled through contrast, which is an indirect proximal cue about distance (there are no resources in the NS for calculating relative movements, depth of focus, and/or relative size combined with any knowledge of absolute size), and (ii). the stability of the constancy (i.e., the formation of veridical percept for the task) comes in a gradual way–as contrast decreases there are fewer formations of veridical percepts for the task, hence fewer avoidances.

20 Parallel processing in multiple retinal pathways and interconnections between them is one of the most important common aspects of the vertebrate retinal organization and functioning (Shigihara and Zeki, 2014). In a very first approximation of the workings of retinal integration processing, the highly complex circuitry of the retina creates a series of parallel pathways from the single layer of photoreceptors. The outer segments of photoreceptors convert (in a process called phototransduction, which involves several catalyzers and feedback loops) incoming light into membrane potential variations, causing thus, synaptic activity in each photoreceptor. In turn, the inner segments of the receptors spatiotemporally integrate the input from phototransduction (through strong modulatory feedback with other cells) into signals that are then transmitted to ganglions that will in turn integrate them in order to convey visual information of the eye to the brain (see Wohrer, 2008 for details).

21 Here we use the concept of neurodynamic structure (NDS) as it has been defined by Barandiaran and Moreno (2006) as “the subset of internal variables and their relationships involved in a certain sensorimotor coupling. An NDS emerges when (for a given time window) we can systematically reduce the dimensionality of the internal operational organization of the NS to explain the behavior of the system.” (p. 177). Thus, we use the term “neurodynamic structure” instead of “neural pattern” to emphasize the specific integrated and higher-level nature of the retinal structure compared to individual retinal cells.

22 Structural similarity has been defined in various ways, but the consensual definition (one that considers and satisfies the important common aspects) is as follows (see O’Brien and Opie, 2004, p. 11 and Isaac, 2013, pp. 688–689): Structure A comprising a set of objects O and a set of relations RO defined on the members of O is structurally similar to structure B comprising a set of components C and a set of relations RC, iff (i) There is a similarity map between AO = f(O, RO) and BC = f(C, RC), such that (ii) This map is a homomorphism induced by a causal process in which the structure of A causally influenced the structure of B, and (iii) The homomorphism results in an (at least partial) structure preservation of the two entities involved so as for at least some objects in O and some relations in RO, there is a one-to-one mapping from O to C and a one-to-one mapping from RO to RC, such that when a relation in RO holds of objects in O, the corresponding relation in RC holds of the corresponding objects in C. In the case of *T. cystophora*, the physical inputs are arrayed across the retina forming an optical image of the object (AV). But as we discussed, this bi-dimensional pattern of excitation on the retina is then projected in different neuronal fields, each one dealing with different aspects [e.g., size, shape, distance–holding in between them various relations (RC)] of the optical information C, and where through various transformations and processing (e.g., filtering) signals–that are related with different visual aspects–are then integrated into the visual signal of the retina BC (i.e., what we call the NDS). Hence, it is the NDS that is the homomorphic structure (BC) of the environmental scene (AO). Considering that the animal responds only to spatial information (and not to mere differences in light intensities), it is sensible to assume that at each stage of processing the components C (sub-patterns of activation in the integrated retinal structure) preserve (at least partially) the respective set of relations (RO) of the object (e.g., an object of a certain size and shape, see also Palmer, 1999).

23 What distinguishes a representational structure from a mere indicator is usually understood as the capacity of the former to represent even in the absence of the stimulus (Ramsey, 2007, pp. 119–20).

24 Even if TC sees the obstacle as a macroscopic object, it definitely cannot see the entire obstacle (i.e., it cannot see all its sides at once). TC is a case of a (cognitive) system whose processes unfold in a purely on-line manner controlling an ongoing, direct interaction with the world. This is in accordance with the suggestion by Gładziejewski and Miłkowski (2017) that structural representations should not be restricted to the domain of off-line cognition (p. 351).

25 A complete defense that structural correspondence is necessary for genuine representation is beyond the scope of this paper. But we suggest it is a well-grounded conjecture according to our analysis.

26 So, the normative standard for evaluating the constancy-bearing NDS is, as argued, that it is used by the animal for a successful obstacle avoidance based on the accuracy of its content obtained on the basis of the exploitable structural similarity of the NDS to its target. It is noted that grounding content in this way requires different explanatory conditions from those used in explaining the dimension of the functional role of the NDS (a requirement also mention by Ramsey, 2016).

27 We will not discuss here the position of the authors that argue that animal vision in general (and especially in the case of phylogenetically older invertebrates) does not need representations; a claim especially maintained by advocates of radical embodiment or/and enactivism (Thompson, 2007; Chemero, 2009; Hutto and Myin, 2013, 2017; Di Paolo et al., 2017). We consider that we have already addressed this objection in section “Obstacle avoidance is mediated by structural representations.”

28 Indeed, each one interaction of *Aglantha digitale* with a certain value of a target object (e.g., a certain strength of water vibration) or with any range of values of a target object, would have its own causal chain of description, part of which will be a different sensory state for each of the values (or range of values) of a target object. Hence, the same sensory state of *A. digitale* cannot be part of different causal chains (e.g., of both escaping and keep swimming leisurely) of the animal’s interaction with the same value of a distal target variable (e.g., strong vibration). Therefore, describing *A. digitale*’s behavior using representational terms for each one of those sensory states would be a characteristic case of a deflationary concept of representation. Schulte (2019) mounts a similar argument to object to liberal representationalism by commenting on a putative representational explanation of the Venus fly trap case. As he says, such ascriptions of representational status violate the explanatory requirement, namely that genuine representational states are those that “they are (a) capable of entering into explanations of behavior qua representational states, i.e., in virtue of their representational properties, and (b) that these explanations cannot be replaced (without loss) by explanations that only appeal to non-representational properties, e.g., explanations that only appeal to the intrinsic, “syntactic” properties of representational states.” (p. 336).
